# Supplementary material for: Comprehensive Annotation and Functional Exploration of MicroRNAs in Lettuce
Source: Front Plant Sci. 2021 Dec 24;12:781836. doi: 10.3389/fpls.2021.781836 (PMC8739914; doi:10.3389/fpls.2021.781836)
Supplement: Supplementary file 3 [file Data_Sheet_9.ZIP › GSM2230752.plot/Lsa-miR396d_Lsat_1_v5_gn_5_141200.1_563_TPlot.pdf]

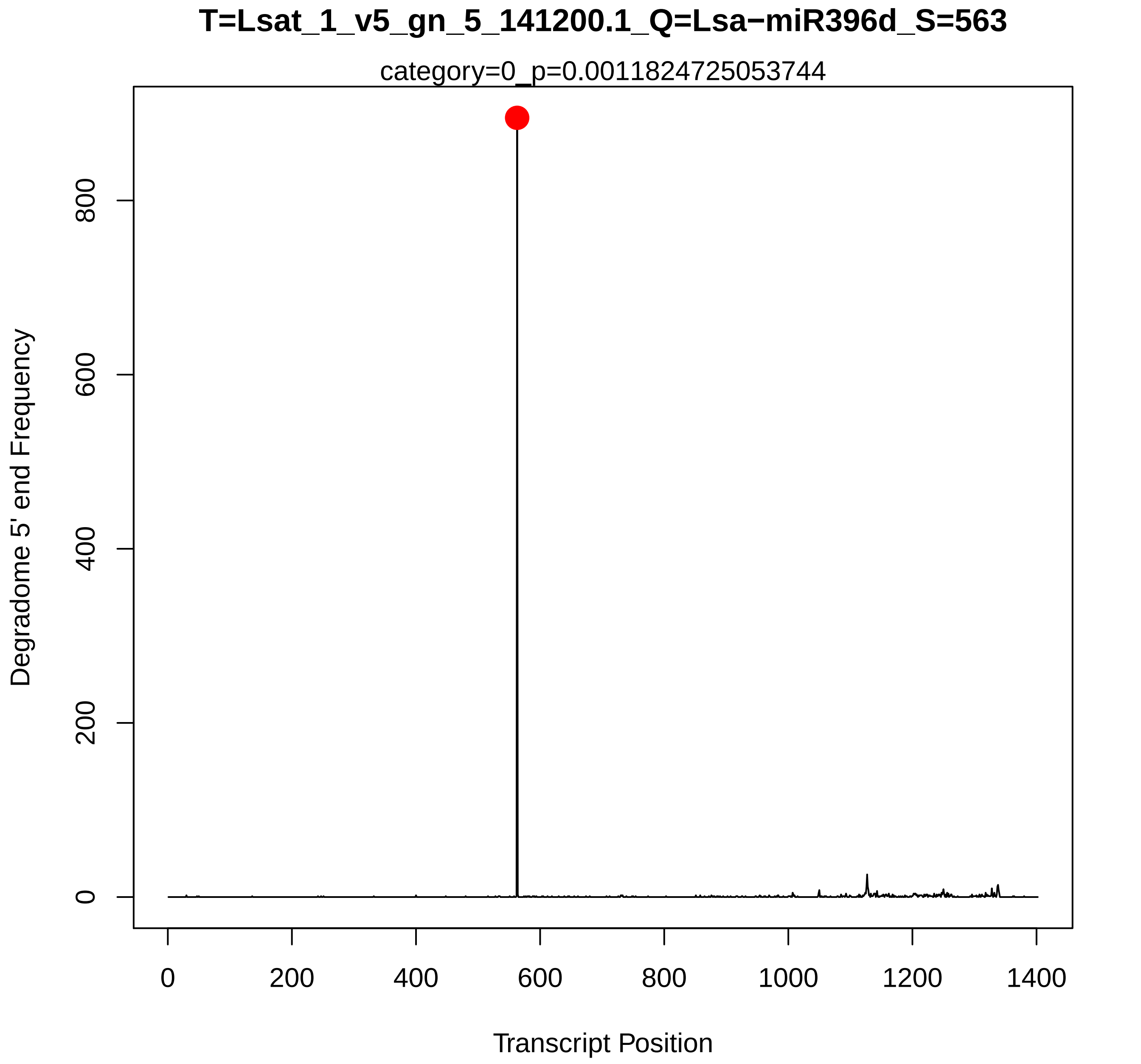
T=Lsat_1_v5_gn_5_141200.1_Q=Lsa−miR396d_S=563
category=0_p=0.0011824725053744
Degradome 5' end Frequency
0
200
400
600
800
0
200
400
600
800
1000
1200
1400
Transcript Position
